# Efficacy and safety of botulinum toxin-A injection in the treatment of premature ejaculation: A systematic review and meta-analysis

**DOI:** 10.1371/journal.pone.0315470

**Published:** 2025-05-30

**Authors:** Yicheng Guo, Fengze Sun, Yini Wang, Yanfei Li, Tianqi Wang, Xiaohong Ma, Jitao Wu

**Affiliations:** 1 Department of Urology, Yantai Yuhuangding Hospital, Qingdao University, Yantai, Shandong, China; 2 The Second Clinical Medical College, Binzhou Medical University, Yantai, China; The University of Tennessee Health Science Center, UNITED STATES OF AMERICA

## Abstract

**Introduction:**

Premature ejaculation (PE) is a common male sexual dysfunction, impacting sexual satisfaction and quality of life. Botulinum toxin-A (BoNT-A), known for its muscle-relaxing properties, has been proposed as a treatment for PE, but its efficacy and safety remain uncertain.

**Objectives:**

To systematically evaluate the efficacy and safety of BoNT-A injection compared to placebo for treating PE.

**Methods:**

A comprehensive search of PubMed, EMBASE, and Cochrane databases was conducted to identify randomized controlled trials comparing BoNT-A and placebo in treating PE. Primary outcomes included intravaginal ejaculatory latency time (IELT) and premature ejaculation profile (PEP), while sexual satisfaction score was a secondary outcome. Mean differences (MD) with 95% confidence intervals (CI) and odds ratios (OR) for adverse events were calculated using a random-effects model.

**Results:**

Three studies were included in the analysis. BoNT-A significantly increased IELT and PEP at the 1-month follow-up (MD = 22.32; 95% CI = 10.83–33.82; P = 0.001 for IELT; MD = 0.91; 95% CI = 0.41–1.42; P < 0.001 for PEP), but no significant differences were observed at 3-month and 6-month follow-ups (P > 0.05). No significant improvements in sexual satisfaction were found (P = 0.32). BoNT-A was associated with a higher incidence of adverse events compared to placebo (OR = 5.90; 95% CI = 1.29–26.89; P = 0.02), but no significant differences were observed for drippling or erectile dysfunction (P > 0.05).

**Conclusion:**

BoNT-A injections may be an effective short-term treatment for PE, significantly improving IELT and PEP at 1-month follow-up. However, the effects appear to diminish over time, and no significant improvement in sexual satisfaction was observed. BoNT-A is associated with a higher rate of adverse events, but does not increase the risk of drippling or erectile dysfunction. Further studies with longer follow-up periods are needed to assess long-term efficacy and safety.

## Introduction

Premature ejaculation (PE) is one of the most common sexual dysfunctions affecting men worldwide, with an estimated prevalence ranging from 20% to 30% of the male population [1,2]. Characterized by a persistent or recurrent pattern of ejaculation occurring within one minute of vaginal penetration, PE significantly impacts sexual satisfaction and quality of life, leading to emotional distress and relationship issues for affected individuals [3,4]. Current treatment options for PE include pharmacological interventions, behavioral therapy, and surgical options, with selective serotonin reuptake inhibitors (SSRIs) and local anesthetics being the most commonly prescribed pharmacotherapies [5]. However, many patients experience limited effectiveness or adverse side effects with these treatments [6].

Botulinum toxin-A(BoNT-A), a neurotoxin that inhibits the release of acetylcholine, has emerged as a novel therapeutic approach for PE [7]. Its ability to relax smooth muscle and potentially modify the neuromuscular control of the pelvic region has led to investigations into its use for extending ejaculatory latency and improving sexual function [8]. Early studies have suggested that BoNT-A injections, particularly those targeting the pelvic floor and prostate area, may increase intravaginal ejaculatory latency time (IELT) and improve overall sexual satisfaction [9,10]. Despite these promising findings, the evidence remains limited and inconsistent, with studies showing variable outcomes and small sample sizes.

Given the growing interest in BoNT-A as a treatment for PE, a systematic evaluation of its efficacy and safety compared to placebo is crucial to determine its potential as a viable therapeutic option. The objective of this meta-analysis is to synthesize the available data from randomized controlled trials (RCTs) to assess the effectiveness of BoNT-A in improving IELT, premature ejaculation profile (PEP), sexual satisfaction, and related outcomes in men with PE, while also evaluating the safety of this intervention. By providing a comprehensive assessment, this study aims to clarify the clinical benefits and risks associated with BoNT-A and inform future treatment strategies for PE.

## Methods

The review protocol was registered with PROSPERO (CRD42024610474; https://www.crd.york.ac.uk/PROSPERO/) following the guidelines outlined in the Preferred Reporting Items for Systematic Reviews and Meta-Analyses (PRISMA).

### Search strategy

We conducted an extensive search of the literature in the PubMed, Embase, and Cochrane databases, following the PRISMA guidelines [11] (S1 Table). The search encompassed articles published from the inception of the databases up until November 2024. Our search strategy was developed based on the PICOS framework (population, intervention, comparators, outcomes, and study design). The primary keywords utilized in our search included “botulinum” and “ejaculation”. Two authors carried out the searches independently according to the predefined strategy, and their findings were subsequently verified against each other. Each article identified was assessed separately by two reviewers, with any discrepancies addressed through consultation with a third researcher. Additionally, relevant references from the studies included in our review were examined as needed.

### Inclusion criteria and data extraction

The inclusion criteria for all articles were as follows:(1) Patient: Male patients aged greater than 18 years, diagnosed with PE according to the definition provided by The International Society for Sexual Medicine (ISSM) in August 2014; (2) intervention: The patients in the trial group were treated with BoNT-A injections; (3) comparator: The patients in the control group were treated with saline injections as a placebo; (4) outcome: The study provided accurate data, including IELT, PEP, sexual satisfaction scores, and complication; and (5) study: All studies were RCTs. Consequently, we excluded animal studies and clinical studies where the control group did not meet the required criteria. Additionally, case reports, review articles, meeting abstracts, and conference reports were also excluded.

### Quality assessment

Two reviewers independently assessed the potential for bias in this study using the Cochrane Risk of Bias (RoB) 2.0 tool [12], concentrating on several areas: the randomization process, deviations from planned interventions, incomplete outcome data, outcome assessment, and the selection of reported results. Any discrepancies between the reviewers were addressed through discussions with a third investigator. Each area was rated as having a “low,” “some concerns,” or “high” risk of bias. The overall risk of bias for each study was based on the highest risk level identified in any of the domains.

### Data extraction and outcome measures

Two authors independently extracted data from the selected studies, compiling details such as the author’s name, publication year, country of study, sample size, treatment regimens and comparators, study duration, outcome measures, adverse events. The primary outcome assessed was IELT, while secondary outcomes included PEP, and sexual satisfaction scores. For studies that did not provide standard deviations (SD), these values were calculated from the available standard errors (SE), confidence intervals (CI), or P values. If none of these metrics were accessible, the SD was estimated based on correlation coefficients obtained from related research. The certainty of evidence for each outcome was assessed using the Grading of Recommendations, Assessment, Development, and Evaluations (GRADE) approach, considering factors such as risk of bias, imprecision, inconsistency, indirectness, and publication bias.

### Statistical analyses

Data analysis for this study was performed using Review Manager version 5.3.0 (Cochrane Collaboration). For binary outcomes, the odds ratio (OR) was calculated, while the mean difference (MD) was used for continuous outcomes, with both reported alongside 95% CI. Cochran’s Q test and the I² statistic were used to evaluate statistical heterogeneity, with heterogeneity defined as I² > 50% or p < 0.05. If no heterogeneity was found, a fixed-effects model was used to pool the effect sizes; if heterogeneity was present, a random-effects model was applied. P < 0.05 was considered statistically significant.

## Results

### Characteristics of included studies

**Fig 1:**
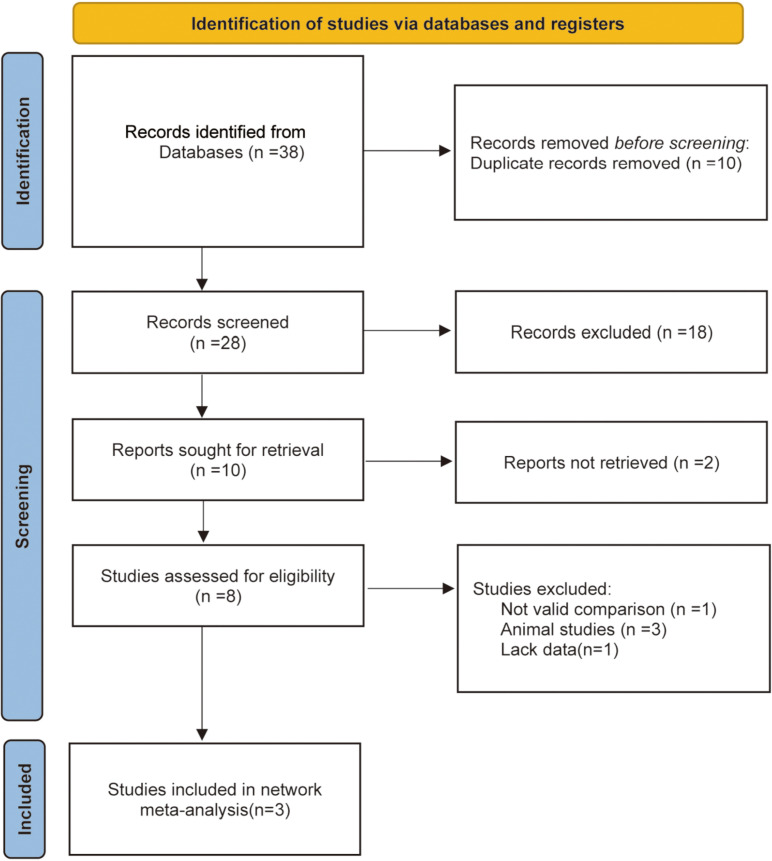
PRISMA flow diagram.

We initially identified 38 articles through our search strategy, but 30 were removed after screening their titles and abstracts. Out of the 8 articles left, 4 were excluded for not meeting the inclusion criteria, and an additional 1 from the remaining 4 were eliminated due to insufficient data. Ultimately, 3 studies were included in our analysis to assess the efficacy and safety of BoNT-A injection in the treatment of premature ejaculation [13–15]. The study selection process is illustrated in Fig 1, with detailed characteristics of these studies provided in Table 1.

**Table 1:** Characteristics of included studies.

Study (years)	Country	Study design	Sample size		Intervention		Duration	Outcome measures	Adverse events
			Trial	Control	Trial	Control			
Almekaty (2024) [13]	Egypt	RCT	29	28	100U botulinum-A toxin in 10 ml saline injection	10 ml saline injection	6 months	IELT, PEP, sexual satisfaction score	Active: mild erectile dysfunction, 1; drippling, 1. Control: urethral bleeding, 1.
Li (2018) [14]	China	RCT	34	35	100U botulinum-A toxin in 10 ml saline injection	10 ml saline injection	4 weeks	IELT, PEP, sexual satisfaction score	Active: mild erectile dysfunction, 4; drippling, 2. Control: not reported
Shaher (2024) [15]	Egypt	RCT	47	45	100U botulinum-A toxin in 10 ml saline injection	10 ml saline injection	6 months	IELT, PEP	Active: infection, 1; drippling, 2; pain,1. Control: not reported

### Risk of bias

**Fig 2:**
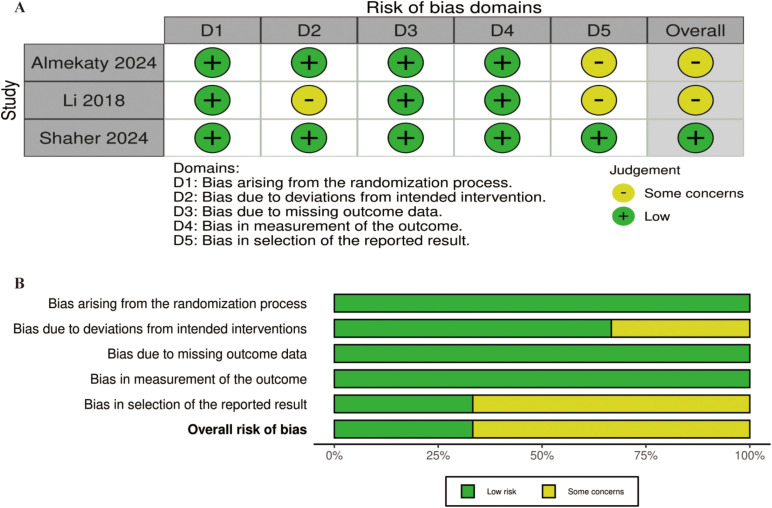
The assessment of risk of bias (RoB). (A) Risk of bias domain for each included study; (B) Summary of risk of bias assessment.

**Fig 3:**
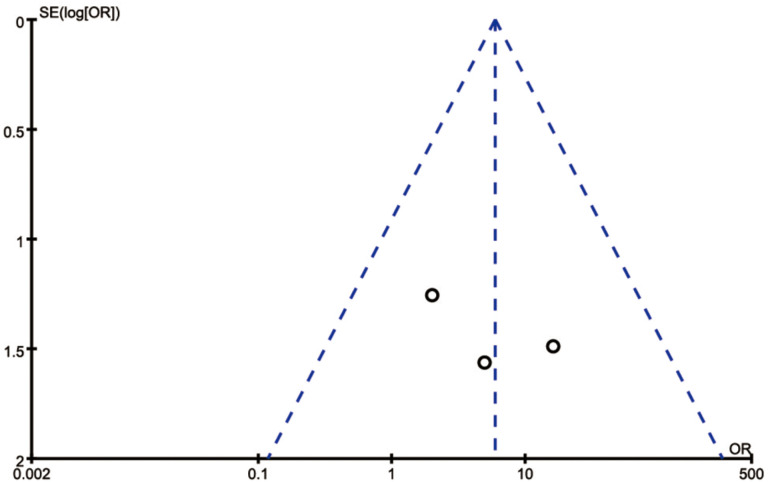
Funnel plot of the articles.

The risk of bias assessment for each study is shown in Fig 2. Out of the included studies, 2 were found to have some concerns, while the remaining 1 was considered to have a low risk. The most common sources of potential bias were related to the randomization process and the selection of reported outcomes. The bias analysis yielded highly symmetrical plots, consisting of 3 squares representing studies that evaluated the efficacy and safety of BoNT-A injection in the treatment of premature ejaculation (Fig 3).

### Assessment of efficacy

According to the GRADE assessment, the certainty of evidence for most outcomes was rated as moderate, while the certainty for some outcomes was downgraded due to imprecision or inconsistency. A detailed summary of the GRADE assessment, including ratings for each outcome and the rationale for downgrading or upgrading the evidence, is provided in S2 Table.

#### IELT.

**Fig 4:**
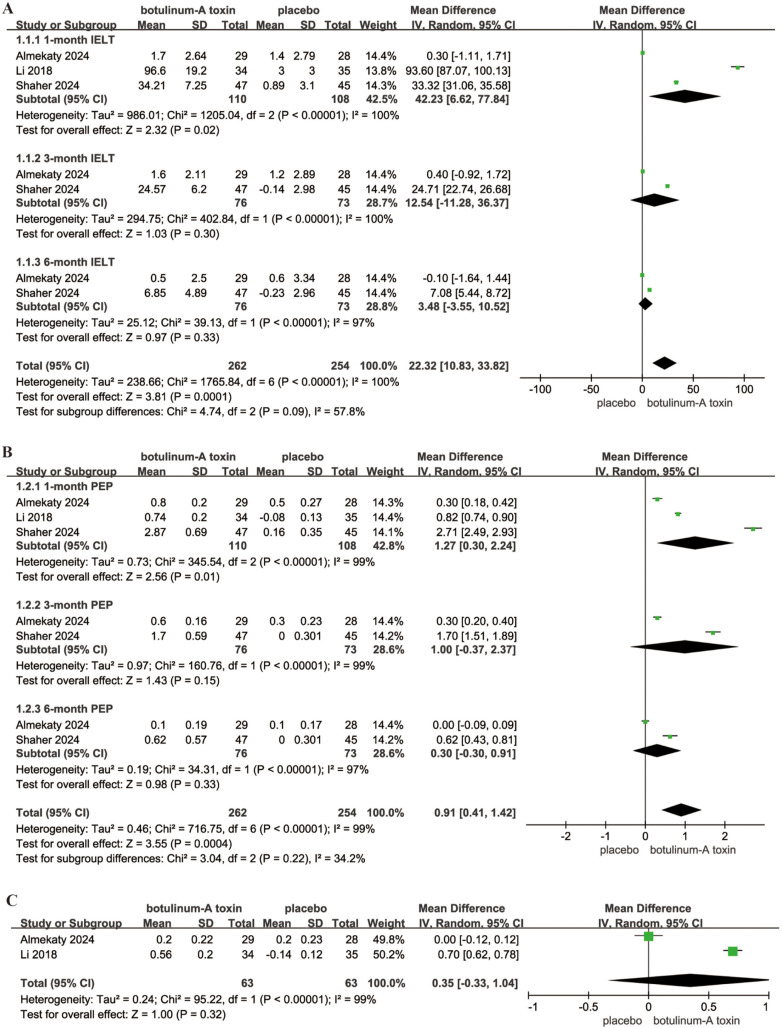
Forest plots showing the pooled results of IELT, PEP and sexual satisfaction scores between BoNT-A and placebo group. (A) IELT; (B) PEP; (C) sexual satisfaction scores. IELT, intravaginal ejaculatory latency time; PEP, premature ejaculation profile; MD, mean difference; CI, confidence intervals.

Three studies provided data on IELT for the comparison between BoNT-A and placebo, with a random-effects model applied to calculate the MD with a 95% CI, given the high heterogeneity among the studies (p < 0.001; I² = 100%). The analysis indicated that BoNT-A significantly increased IELT compared to the placebo group (MD = 22.32; 95% CI = 10.83–33.82; P = 0.0001). Specifically, at the 1-month follow-up, IELT was significantly prolonged in the BoNT-A group (MD = 42.23; 95% CI = 6.62–77.84; P = 0.02). However, no significant differences were observed at the 3-month (P = 0.30) and 6-month (P = 0.33) follow-ups (Fig 4A), suggesting that the effect of BoNT-A may diminish over time.

#### PEP.

Three studies provided data on PEP for comparing BoNT-A and placebo, with a random-effects model used to calculate the effects, due to the considerable heterogeneity among the studies (p < 0.001; I² = 100%). The results showed that BoNT-A notably increased PEP compared to placebo (MD = 0.91; 95% CI = 0.41–1.42; P = 0.0004). In particular, PEP at the 1-month follow-up was significantly longer in the BoNT-A group (MD = 1.27; 95% CI = 0.3–2.24; P = 0.01). However, no significant differences were observed at the 3-month (P = 0.15) and 6-month (P = 0.33) follow-ups (Fig 4B), suggesting that the effect of BoNT-A may decrease over time.

### Sexual satisfaction score

Two studies reported data on sexual satisfaction scores for comparing BoNT-A with placebo. Only the data from the first month of follow-up were included in the analysis. Due to substantial heterogeneity among the studies (p < 0.001; I² = 99%), a random-effects model was used to calculate the efficacy. The results showed no significant difference in sexual satisfaction between two groups at the 1-month follow-up (P = 0.32) (Fig 4C), indicating that BoNT-A did not significantly improve sexual satisfaction compared to placebo.

### Assessment of safety

#### Adverse events.

**Fig 5:**
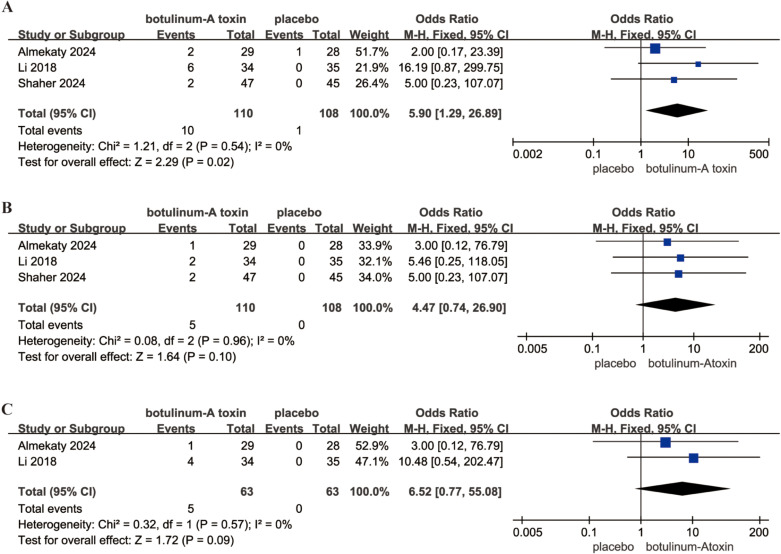
Forest plots showing the pooled results of adverse events, drippling, and mild ejaculation dysfunction. (A) adverse events; (B) drippling; (C) mild ejaculation dysfunction. MD, mean difference; CI, confidence intervals.

Three studies, comprising a total of 218 participants (110 in the intervention group and 108 in the control group), reported data on adverse events comparing BoNT-A with placebo. Due to low heterogeneity among the studies (p = 0.54; I² = 0), a fixed-effects model was used to calculate the safety. The results showed that the OR was 5.90 and the 95% CI was 1.29–26.89 (P = 0.02) (Fig 5A), which meant that BoNT-A injection led to more adverse events than did placebo.

#### Drippling.

A total of 218 participants (110 in the intervention group and 108 in the control group) from three studies provided data on drippling to compare BoNT-A with placebo. Given the low heterogeneity among the studies (p = 0.96; I² = 0), a fixed-effects model was employed to assess safety. The results showed no significant difference in drippling between two groups (P = 0.1) (Fig 5B), indicating that BoNT-A injection did not significantly increase the possibility of drippling than placebo.

#### Mild erectile dysfunction.

Data on erectile dysfunction were provided by two studies, including a total of 126 participants (63 in the intervention group and 63 in the control group), to compare BoNT-A with placebo. With the low heterogeneity between the studies (p = 0.57; I² = 0), a fixed-effects model was used to evaluate safety. The findings revealed no significant difference in erectile dysfunction between the two groups (P = 0.09) (Fig 5C), suggesting that BoNT-A injection did not notably increase the risk of mild erectile dysfunction compared to placebo.

## Discussion

PE remains one of the most common sexual disorders among men, significantly impacting quality of life [16]. Various pharmacological treatments have been explored for the management of PE, with SSRIs and α-adrenergic receptor antagonists being the most commonly used [17]. Recently, BoNT-A has been considered as a potential treatment option for PE. In addition to its medical applications, BoNT-A is widely used for cosmetic purposes, particularly for the treatment of facial wrinkles and signs of aging [18]. Its ability to temporarily paralyze the muscles responsible for wrinkle formation has made it a popular non-surgical intervention for facial rejuvenation [19]. Despite its widespread use and generally favorable safety profile, BoNT-A injections are not without side effects. The most commonly reported adverse effects include local reactions such as bruising, swelling, and pain at the injection site, as well as headache and drooping in some individuals [20]. Serefoglu et al. [21] proposed that BoNT-A could be injected into the muscle to inhibit its contraction in order to prolong ejaculation, we systematically evaluated the efficacy and safety of BoNT-A injections compared to placebo in treating PE.

Our findings demonstrate that BoNT-A may offer short-term benefits in managing PE, particularly in improving IELT and PEP. Specifically, our analysis revealed that BoNT-A significantly increased IELT and PEP at the 1-month follow-up. However, the effects appeared to diminish over time, with no significant differences observed at the 3-month and 6-month follow-ups. This decline in efficacy over time aligns with previous studies, which have noted that the effects of BoNT-A injections are typically temporary [22,23], likely due to the gradual clearance of the toxin from the body [24,25]. While this limitation is important to consider, the short-term benefits of BoNT-A may still make it a valuable option for patients seeking immediate relief from PE.

In terms of sexual satisfaction, our analysis showed no significant difference between two groups. Despite the improvements in IELT and PEP, which are key physiological markers of PE [26], BoNT-A did not appear to significantly improve sexual satisfaction. This finding highlights the complex nature of sexual satisfaction, which is influenced not only by physical factors such as ejaculatory control, but also by psychological, emotional, and relational aspects [27]. Therefore, while BoNT-A may address certain physiological components of PE, it may not fully improve the broader psychological experience of sexual satisfaction, emphasizing the need for a more comprehensive treatment approach that incorporates both physical and psychological factors.

Regarding safety, our analysis found that BoNT-A injections were associated with a higher incidence of adverse events compared to placebo. The increased frequency of adverse events in our analysis proposes that careful patient selection and monitoring are essential when considering BoNT-A as a treatment for PE. Interestingly, our analysis did not find significant differences between the BoNT-A and placebo groups in terms of drippling or mild erectile dysfunction, implying that BoNT-A did not exacerbate these specific adverse effects. Meanwhile, Abdelrahman et al. [25] found that systemic toxicity from BoNT-A injections did not occur in any study population, nor were there any other severe local side effects. These results are promising, as they show that the adverse effects associated with BoNT-A may be relatively limited in the context of PE treatment, particularly when compared to other treatments that may cause more pronounced sexual side effects, such as α-adrenergic receptor antagonists.

Our study contributes to the growing body of literature on the use of BoNT-A for PE, providing a comprehensive review of its efficacy and safety. However, several limitations should be considered when interpreting our findings. The certainty of evidence for most outcomes was moderate, suggesting that the findings are relatively robust, though some limitations exist. First, the high heterogeneity observed in some of the analyses, particularly regarding IELT and PEP, suggests that the included studies varied considerably in terms of study design, patient characteristics, and intervention protocols. Although a random-effects model was employed to account for this heterogeneity, the variability between studies should be noted as a potential limitation. Second, the relatively short follow-up periods in most of the included studies limit the ability to assess the long-term efficacy of BoNT-A for PE. Future studies with longer follow-up durations are necessary to evaluate whether BoNT-A provides sustained benefits or if repeated treatments are required to maintain its effects. Additionally, the limited number of studies included in this meta-analysis may have reduced the statistical power of our findings, especially for some secondary outcomes. The variability in sample sizes and study methodologies also poses challenges in drawing definitive conclusions. Most of the included studies were conducted in specific regions, which may limit the generalizability of the findings to other populations. Future research should aim to include larger and more diverse cohorts to enhance the generalizability of the results.

## Conclusion

In conclusion, this meta-analysis provides evidence supporting the short-term efficacy of BoNT-A in improving certain physiological markers of premature ejaculation, such as IELT and PEP. However, the effects appear to diminish over time, and no significant improvements are found in sexual satisfaction. The safety profile of BoNT-A suggests an increased risk of adverse events compared to placebo, although no significant differences were found in terms of drippling or mild erectile dysfunction. Given these findings, BoNT-A may be a viable option for patients seeking rapid relief from PE, but further studies with longer follow-up periods are needed to assess its long-term benefits and safety. Moreover, a more comprehensive treatment approach that addresses both physical and psychological aspects of PE should be considered to optimize patient outcomes.

## Supporting information

S1 Table PRISMA guidelines. (DOCX)

S2 Table GRADE table. (DOCX)

S1 File Minimal Data Set. (XLSX)

S2 File Studies Identified in the Literature Search. (XLSX)

S3 File ROB2 risk-of-bias. (XLSX)
